# Determination of Normal Ranges of Shock Index and Other Haemodynamic Variables in the Immediate Postpartum Period: A Cohort Study

**DOI:** 10.1371/journal.pone.0168535

**Published:** 2016-12-20

**Authors:** Hannah L. Nathan, Kate Cottam, Natasha L. Hezelgrave, Paul T. Seed, Annette Briley, Susan Bewley, Lucy C. Chappell, Andrew H. Shennan

**Affiliations:** Women’s Health Academic Centre, King’s College London, London, United Kingdom; University of North Carolina at Chapel Hill, UNITED STATES

## Abstract

**Objective:**

To determine the normal ranges of vital signs, including blood pressure (BP), mean arterial pressure (MAP), heart rate (HR) and shock index (SI) (HR/systolic BP), in the immediate postpartum period to inform the development of robust obstetric early warning scores.

**Study Design:**

We conducted a secondary analysis of a prospective observational cohort study evaluating vital signs collected within one hour following delivery in women with estimated blood loss (EBL) <500ml (316 women) delivering at a UK tertiary centre over a one-year period. Simple and multiple linear regression were used to explore associations of demographic and obstetric factors with SI.

**Results:**

Median (90% reference range) was 120 (100–145) for systolic BP, 75 (58–90) for diastolic BP, 90 (73–108) for MAP, 81 (61–102) for HR, and 0.66 (0.52–0.89) for SI. Third stage Syntometrine^®^ administration was associated with a 0.03 decrease in SI (p = 0.035) and epidural use with a 0.05 increase (p = 0.003). No other demographic or obstetric factors were associated with a change in shock index in this cohort.

**Conclusion:**

This is the first study to determine normal ranges of maternal BP, MAP, HR and SI within one hour of birth, a time of considerable haemodynamic adjustment, with minimal effect of demographic and obstetric factors demonstrated. The lower 90% reference point for systolic BP and upper 90% reference point for HR correspond to triggers used to recognise shock in obstetric practice, as do the upper 90% reference points for systolic and diastolic BP for obstetric hypertensive triggers. The SI upper limit of 0.89 in well postpartum women supports current literature suggesting a threshold of 0.9 as indicating increased risk of adverse outcomes.

## Introduction

The UK and Ireland Confidential Enquiries into Maternal Deaths and Morbidity Report of 2009–2012 (Mothers and Babies: Reducing Risk through Audits and Confidential Enquiries across the UK (MBRRACE)) highlighted that failures by healthcare professionals to immediately recognise and act on signs of life-threatening conditions, including haemorrhage, severe pre-eclampsia and sepsis, may have contributed to potentially avoidable direct maternal deaths [1]. The report emphasised the importance of routine measurement of vital signs and recommended the use of modified early obstetric warning score (MEOWS) charts in all pregnant and postpartum women to aid more timely recognition of compromise [1]. According to the 2014 systematic review of causes of maternal mortality by the World Health Organisation, obstetric haemorrhage, hypertensive disorders and sepsis contribute to approximately 50% of maternal deaths worldwide. All are associated with changes in vital signs, including blood pressure (BP) and heart rate (HR) [2]. Obstetric haemorrhage is the leading cause of death (27.1% of all deaths, 95% CI 19.9 to 36.2%) [2] with the majority occurring during labour, delivery and the immediate postpartum period [3]. Failure to recognise deterioration contributes to many of these avoidable deaths [4].

Changes in conventional vital signs into the abnormal range are late markers of compromise [5–6]. Relying solely on changes in BP and HR as individual parameters may delay vital interventions in women with postpartum haemorrhage (PPH), defined as estimated blood loss (EBL) of 500ml or more, and may contribute to avoidable mortality and morbidity.[6] Shock Index (SI), the ratio of HR to systolic BP, has been proposed as an alternative measure of early compromise and compares favourably to conventional vital signs in predicting risk of adverse clinical outcomes in women with PPH [7]. Despite this, the normal ranges of SI and conventional vital signs in the immediate postpartum period (i.e. within one hour of delivery) have not yet been adequately defined.

The aim of this study was to determine the normal range of maternal SI, in addition to BP, mean arterial pressure (MAP) and HR within an hour of birth in women with normal blood loss, in order to enable subsequent exploration of its use as an early warning system to identify the deteriorating woman.

## Materials and Methods

The case records of all women chosen for the control arm within a prospective weighted-sample cohort study were examined i.e. women with blood loss at delivery within normal limits. The purpose of the original study was to determine risk factors for PPH. Women delivering at a UK maternity unit with an EBL of less than 500mls were chosen as a one-in-twelve random sample (for comparison against women with PPH delivering over a one-year period) and the data used in this analysis. At this unit, blood loss at caesarean section was calculated through weighing of swabs and drapes and 10% of documented data on blood loss values underwent rigorous validation involving an independent expert obstetrician. It is possible that a minority of women with blood losses above the normal threshold have been included in the cohort (unlikely to be overt haemorrhages). As these women were identified as having ‘normal’ blood losses and were therefore treated as clinically ‘normal’, our results remain valid and valuable. The original study was approved by the South East multi-centre research ethics committee [8]. Individual informed consent was not required for this observational study, as data collection did not directly involve contact with women. All women with normal blood loss following delivery (including, for example, those with multi-fetal pregnancies or caesarean delivery) were included, to provide a representative sample of women. The first BP and HR values recorded in clinical practice within the hour following delivery were used in the analysis. Method of measurement varied according to clinician preference but automated devices with digital displays were routinely available. In women who underwent caesarean section or instrumental delivery, the durations of the first, second and third stages may have been iatrogenically altered. Third stage policy was to offer uterotonic agents for active third stage prophylaxis as then recommended by the National Institute for Health and Care Excellence (NICE) but physiological third stage was also practiced [9].

Data analysis was conducted using Stata version 11.2 (StataCorp, College Station, Texas). Median, lower and upper quartiles and 90% reference ranges were calculated for BP, HR, MAP and SI.

Using simple and multiple linear regression analysis, the association with SI of nineteen predefined demographic and obstetric factors was investigated, based on prior examination of the literature and sufficient data on the variable: age, parity, ethnicity, weight, height, body mass index, multi-fetal pregnancy, duration of first stage, second stage and third stage of labour, mode of delivery (vaginal or caesarean section), Syntometrine^**®**^ administration for the third stage, oxytocin administration for the third stage, epidural anaesthetic, spinal anaesthetic, temperature during labour, anaemia, gestational hypertension, pre-eclampsia. For variables with strong non-normal distributions (duration of first, second and third stage of labour and EBL) medians and IQR were calculated.

## Results

384 control women with an EBL <500ml were identified. There were 68 exclusions due to missing vital signs documentation (n = 24) and delivery-observation time greater than one hour (n = 44). 316 women were included in statistical analysis, allowing estimation of the 95^th^ centile to within 0.17 of a standard deviation [10]. Participant characteristics are shown in Table 1.

**Table 1:** Participant Characteristics. Values are given as number (percentage), mean (SD) or median [IQR].

Characteristics	Participants (n = 316)
Mean (SD) age at delivery, years	30.7 (5.6)
**Parity at trial entry n (%)**	
0	163 (51.6)
1	85 (26.9)
2	42 (13.3)
3+	26 (8.2)
**Ethnicity n (%)**	
White	156 (49.9)
Black	89 (28.2)
Asian	39 (12.3)
Other	32 (10.1)
Mean (SD) Body Mass Index, kg/m^2^	24.5 (5.2)
Multi-fetal pregnancy n (%)	6 (1.9)
Median [IQR] duration of 1^st^ Stage, minutes	270 [140, 475]
Median [IQR] duration of 2^nd^ Stage, minutes	30 [10, 90]
Median [IQR] duration of 3^rd^ Stage, minutes	8 [5,14]
**Mode of delivery n (%)**	
Vaginal delivery (including instrumental)	278 (88.0)
Caesarean delivery	38 (12.0)
Syntometrine^®^ administration in the 3^rd^ stage n (%)	206 (65.2)
Oxytocin administration in the 3^rd^ stage n (%)	66 (20.9)
Physiological management of 3^rd^ stage n (%)	44 (13.9)
Epidural anaesthetic n (%)	69 (21.8)
Spinal anaesthetic n (%)	33 (10.4)
Mean (SD) temperature during labour,°C	36.7 (0.49)
Anaemia (Hb <10.5 g/ml in pregnancy) n (%)	43 (13.6)
Gestational hypertension n (%)	8 (2.5)
Pre-eclampsia n (%)	4 (1.3)
Median [IQR] blood loss, ml	300 [200, 400]
Median [IQR] time from delivery to SI reading, minutes	8 [5, 13]

**Fig 1:**
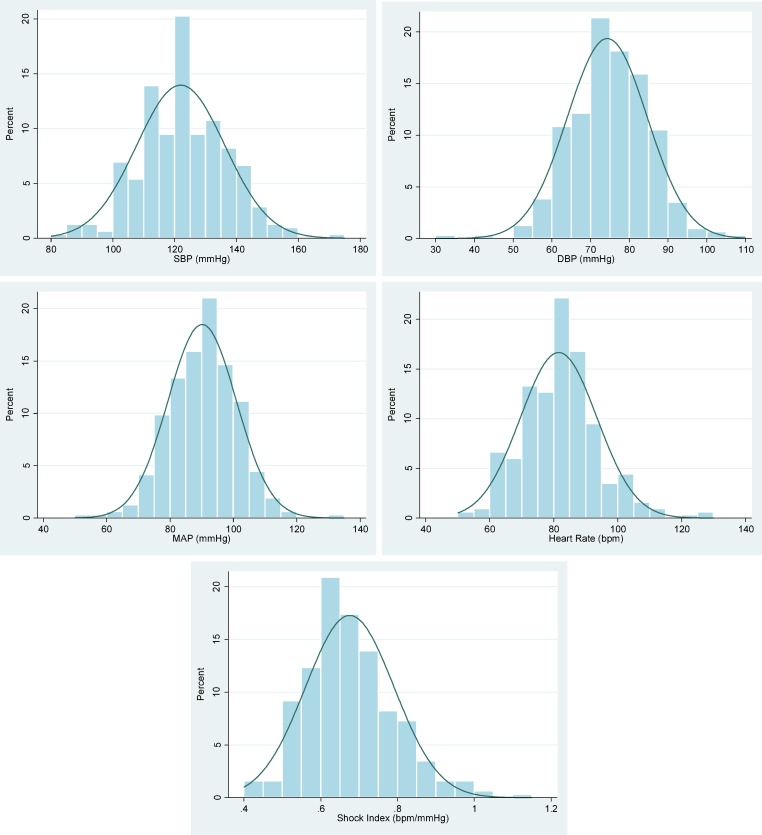
Histograms of distribution of BP, MAP, HR and SI values (with superimposed normal distribution curves). SBP: systolic blood pressure, DBP: diastolic blood pressure, MAP: mean arterial pressure, HR: heart rate, SI: shock index.

Median, lower and upper quartile and 90% reference ranges for systolic BP, diastolic BP, MAP, HR and SI are shown in Table 2. The 90% reference range of SI is 0.52–0.89. Histograms for systolic BP, diastolic BP, MAP, HR and SI, including superimposed normal distribution curves, are shown in Fig 1. The similarity between the histograms and the normal distribution curve confirms that tests based on the normal distribution are appropriate.

**Table 2:** Median, lower and upper quartile and 90% reference ranges for systolic blood pressure, diastolic blood pressure, mean arterial pressure, heart rate and shock index

	Median	Lower quartile	Upper quartile	90% Reference Range
**Systolic BP** (mmHg)	120	111	131	100–145
**Diastolic BP** (mmHg)	75	68	80	58–90
**Mean arterial pressure** (mmHg)	90	83	97	73–108
**Heart rate** (bpm)	81	74	88	61–102
**Shock index**	0.66	0.60	0.74	0.52–0.89

Significant associations (using simple then multiple linear regression methods) were noted between SI and third stage Syntometrine^**®**^ and epidural use (Table 3). SI decreased by 0.03 with third stage use of Syntometrine^**®**^, predominantly due to HR decrease rather than systolic BP rise, equating to median SI decreasing from 0.66 to 0.63. SI increased by 0.05 with epidural use, due to HR increase rather than BP decrease, equating to median SI increase from 0.66 to 0.71. No other factors retained significance in the multiple regression analysis. In this cohort of normal women, length of labour was relatively short (compared to duration for an unselected population of labouring women) and this may have been due to exclusion of prolonged labour in women who went on to have postpartum haemorrhage. The median [IQR] time from delivery to SI reading was 8 [5, 13]. The mean SI was 0.63 prior to delivery of placenta, 0.68 at the same time as delivery of placenta, and 0.68 following delivery of placenta. An observed difference in SI of 0.05 is clinically unimportant.

**Table 3:** Association between shock index and demographic and obstetric factors. Change in mean shock index associated with a 1-unit change in each predictor is shown.

	Change in mean SI	Confidence Interval	P-value
**Simple Linear Regression Analysis**			
Age (in decades)	-0.01	-0.03, 0.01	0.332
Parity			
1	0.001	-0.029, 0.032	0.938
2	-0.013	-0.053, 0.026	0.507
3+	-0.032	-0.080, 0.016	0.195
Ethnicity			
Black	0.017	-0.014, 0.047	0.281
Asian	0.030	-0.011, 0.071	0.148
Other	0.001	-0.043, 0.045	0.968
Weight (kg)	0.000	-0.001, 0.001	0.903
Height (cm)	-0.002	-0.004, 0.000	0.052
BMI	0.001	-0.001, 0.004	0.380
Multiple Pregnancy	-0.093	-0.186, 0.0001	0.050
Duration of 1^st^ Stage	0.003	-0.002, 0.003	0.816
Duration of 2^nd^ Stage	-0.001	-0.009, 0.007	0.818
Duration of 3^rd^ Stage	-0.001	-0.014, 0.012	0.890
Mode of Delivery (caesarean vs vaginal)	0.011	-0.028, 0.051	0.572
Syntometrine^®^ administration in the 3^rd^ stage	-0.029	-0.055, -0.002	0.035
Oxytocin administration in 3^rd^ stage	0.031	0.000, 0.062	0.053
Epidural anaesthetic	0.046	0.0159, 0.770	0.003
Spinal anaesthetic	-0.160	-0.579, 0.026	0.451
Temperature during labour	-0.004	-0.031, 0.022	0.741
Anaemia (last recorded in pregnancy)	-0.003	-0.143, 0.009	0.619
Gestational hypertension	0.011	-0.710, 0.920	0.800
Pre-eclampsia	-0.058	-0.172, 0.056	0.319
**Multivariable Regression Analysis**			
Epidural anaesthetic	-0.030	-0.057, -0.002	0.036
Syntometrine^®^ administration in 3^rd^ stage	0.047	0.016, 0.079	0.003

## Discussion

This study is original, defining the normal range of BP, HR, MAP and SI within the first hour after birth. Previous studies evaluating postpartum haemodynamic changes have proposed normal ranges for vital signs. However, all have used time points beyond the first hour [11–14], despite recognition in the World Health Organization Post Natal Guidelines of the need for appropriate surveillance to start within the first hour after birth [15]. This study defines the normal ranges for maternal BP, HR, and MAP in the first hour postpartum, all of which are used routinely to assess the haemodynamic status of women following birth. The lower 90% reference range for systolic BP (100mHg) corresponds with the amber lower threshold of systolic BP (100 mmHg) and the upper 90% reference range for HR (102 bpm) corresponds with the amber upper threshold of HR (100 bpm) for the recognition of shock on the currently recommended MEOWS chart [16]. The upper 90% reference ranges for systolic and diastolic BP (145 mmHg and 90 mmHg, respectively) correspond well with the early warning chart amber hypertensive triggers (150 mmHg and 90 mmHg) [16]. It is important to define a normal range as pregnant and recently pregnant women may decompensate relatively late following haemorrhage and sepsis compared to other adults.

The strength of this study is that it addresses the need for evidence-based vital sign reference ranges to guide the monitoring of postpartum women worldwide and subsequent timely intervention. The multi-ethnic population studied is a further strength of the study increasing the generalisability of the findings.

It is recognised that the values may not be applicable beyond the first hour and that results may be influenced by the particular setting (i.e. maternity unit in a high-income country). Considering the dramatic haemodynamic changes during pregnancy and postpartum, more precise prospective studies are ongoing to define the normal ranges of BP, HR, MAP and SI at different gestations, intrapartum and in the later postpartum period, and in low-resource settings.

As a secondary analysis of a prospective observational study, the accuracy of vital sign values recorded depended on the BP devices used and the healthcare provider measuring and recording the vital signs. Fig 1 highlights the issue of digit preference, with a striking proportion of values recorded as systolic BP of 120mmHg and HR of 80bpm (standard values), potentially confounding the results. Future work should collect vital signs prospectively in women immediately postpartum, using automated devices that have been validated for use in pregnancy, to minimise user error and improve accuracy.

Clinical assessment of postpartum haemorrhage includes accurate estimation of blood loss and timely measurement of vital signs. It is well recognised that, despite being the most commonly used technique, visual estimation of blood loss frequently underestimates [17]. As a consequence, appropriate use of vital signs is critical, as reflected in The UK and Ireland Confidential Enquiries into Maternal Deaths and Morbidity Report of 2009–2012 (Mothers and Babies: Reducing Risk through Audits and Confidential Enquiries across the UK (MBRRACE)). SI was first proposed as an early marker of haemodynamic instability in non-obstetric shock in 1967 [18]. It has been studied extensively in non-specific shock [19–21], trauma [22–28], and sepsis [29] as an earlier identifier of circulatory shock than conventional vital signs. In a non-obstetric population the normal range has been defined and validated as 0.5 to 0.7 [19, 21, 30–31], with a SI >0.9 indicating increased risk of mortality and morbidity [27]. In pregnancy, studies have suggested an upper limit of normal of 0.7 [5], 0.81 [32], and 0.85 [33], based on prediction of ruptured ectopic pregnancy, and 0.9 [7], based on prediction of ICU admission in women with PPH. Only one study has attempted to define SI normal range, in a retrospective case-control analysis of women experiencing PPH [34]. In their control group of only 50 women with normal blood loss, mean SI was 0.74 with a range of 0.4–1.1, ten minutes following birth. Although the methodology was unclear, a normal range of 0.7–0.9 for an obstetric population was proposed from these data. This previous work is supported by the upper limit of normal of 0.9 in the current cohort (0.52–0.89). Both are higher than the upper limit of normal of 0.7 in a non-obstetric population. The difference can be explained by the haemodynamic changes of pregnancy and delivery, namely an increase in resting HR, which is often further increased during the immediate postpartum period owing to pain and exertion.

Pregnancy involves significant haemodynamic changes [35]. As the placenta is delivered, auto-transfusion results in cardiac output increasing to a maximal 80% above pre-pregnancy values [35]. It is in this dynamic intrapartum and immediate postpartum period that haemorrhage and sepsis is most prevalent, compensatory mechanisms can mask hypovolaemia and subtle changes in vital signs measurement may alert healthcare providers prior to severe compromise, thereby limiting short- and longer-term maternal morbidity.

The UK Confidential Enquiry report into Maternal Death repeatedly asserts the importance of vital signs measurement and recommends early warning charts for all antenatal and postnatal admissions despite lack of evidence of clinical and cost-effectiveness [1]. These charts are held as a beacon of good practice internationally and have been widely emulated in other countries, including USA [36] and South Africa [37]. However, a standardised obstetric-specific chart does not exist; there is no agreed combination of vital signs and trigger thresholds for vital signs have not been adequately determined [1]. One example of a early warning chart (from the Confidential Enquiry in 2007) has been adopted by hospitals in the UK and elsewhere, but was only validated in 2012, demonstrating adequate predictive value, but suggesting further refinements were required [16]. There has been very little research since about the vital signs thresholds that specifically indicate decompensation in an obstetric population, a particular concern considering the haemodynamic changes of pregnancy and postpartum. Our findings support current early warning thresholds in the immediate postpartum period.

Demographic and iatrogenic influences impacting on the haemodynamic physiology of pregnancy will influence vital signs. In this setting (a UK maternity unit), uterotonics are routinely administered as the baby is delivered to prevent PPH. In low-resource settings, active management of the third stage is used less frequently, due to limited availability of uterotonic medication. Syntometrine^**®**^, a combination of ergometrine and oxytocin, is the most commonly used preparation in high-income countries but is usually contraindicated in those with hypertension, owing to the hypertensive effects of ergometrine [38]. Oxytocin alone is often used in those with Syntometrine^**®**^ contraindications and also has important side-effects, including hypotension and tachycardia [39]. In women receiving either epidural or spinal analgesia or anaesthesia, blood pressure can be reduced due to sympathetic block. These data show that Oxytocin alone had no significant effect on SI. The observed decrease in SI with Syntometrine^**®**^ use was largely due to a decrease in HR, rather than the anticipated increase in SBP. Epidural use was associated with an SI increase, again, largely due to changes in HR, rather than the expected hypotensive effects of epidural use [40]. Although statistically significant, syntometrine use and epidural use did not move the median SI outside of the normal range and are likely of minimal clinical importance. Spinal anaesthetic use was not associated with a change in SI, likely due to the timing of vital signs used for analysis; the vasodilatory effects of spinal anaesthesia are most marked at administration prior to delivery, rather than at the time of vital sign measurement postpartum.

Study participants were defined by having normal outcome blood loss and there was a low incidence of hypertension and anaemia. We have previously shown that these variables have little influence on SI in postpartum haemorrhage. In women with postpartum haemorrhage, confounding factors including spinal and epidural use, and Syntometrine for the management of the third stage, have previously been shown to have negligible effects on SI [7]. Considering the minimal effect of demographic and obstetric factors on SI in women with postpartum haemorrhage and women with normal blood loss, the normal range of SI does not need to be altered according to the presence or absence of these factors, despite their prevalence differing between settings e.g. well-resourced and low-resourced settings.

A robust evidence-base underpinning the recommended and widely used MEOWS chart does not exist. Studies are required to establish the impact, risks and benefits and cost-effectiveness of MEOWS charts. SI has previously been shown to be a consistently strong predictor of a range of adverse clinical outcomes in women with PPH and has the advantage of being automatable [7]. This work defines the normal range of maternal SI in the first hour after birth. Future work should focus on prospective evaluation of SI in uncomplicated pregnancy, obstetric haemorrhage and sepsis. Studies should also consider the impact of gestational age and stage of labour on SI and assess the added value of incorporating SI into MEOWS charts.

In conclusion, this study is the first to determine the normal range of SI of 0.52–0.89 in the first hour postpartum as well as the normal range of BP, HR and MAP. These findings may have implications for guiding intervention immediately postpartum.
